# Neural Correlates of Four Broad Temperament Dimensions: Testing Predictions for a Novel Construct of Personality

**DOI:** 10.1371/journal.pone.0078734

**Published:** 2013-11-13

**Authors:** Lucy L. Brown, Bianca Acevedo, Helen E. Fisher

**Affiliations:** 1 Department of Neurology, Einstein College of Medicine, Bronx, New York, United States of America; 2 Department of Psychological and Brain Sciences, University of California Santa Barbara, Santa Barbara, California, United States of America; 3 Department of Anthropology, Rutgers University, New Brunswick, New Jersey, United States of America; Catholic University of Sacred Heart of Rome, Italy

## Abstract

Four suites of behavioral traits have been associated with four broad neural systems: the 1) dopamine and related norepinephrine system; 2) serotonin; 3) testosterone; 4) and estrogen and oxytocin system. A 56-item questionnaire, the Fisher Temperament Inventory (FTI), was developed to define four temperament dimensions associated with these behavioral traits and neural systems. The questionnaire has been used to suggest romantic partner compatibility. The dimensions were named: Curious/Energetic; Cautious/Social Norm Compliant; Analytical/Tough-minded; and Prosocial/Empathetic. For the present study, the FTI was administered to participants in two functional magnetic resonance imaging studies that elicited feelings of love and attachment, near-universal human experiences. Scores for the Curious/Energetic dimension co-varied with activation in a region of the substantia nigra, consistent with the prediction that this dimension reflects activity in the dopamine system. Scores for the Cautious/Social Norm Compliant dimension correlated with activation in the ventrolateral prefrontal cortex in regions associated with social norm compliance, a trait linked with the serotonin system. Scores on the Analytical/Tough-minded scale co-varied with activity in regions of the occipital and parietal cortices associated with visual acuity and mathematical thinking, traits linked with testosterone. Also, testosterone contributes to brain architecture in these areas. Scores on the Prosocial/Empathetic scale correlated with activity in regions of the inferior frontal gyrus, anterior insula and fusiform gyrus. These are regions associated with mirror neurons or empathy, a trait linked with the estrogen/oxytocin system, and where estrogen contributes to brain architecture. These findings, replicated across two studies, suggest that the FTI measures influences of four broad neural systems, and that these temperament dimensions and neural systems could constitute foundational mechanisms in personality structure and play a role in romantic partnerships.

## Introduction

Four suites of behavioral traits have been extracted from the literature, each associated with one of four broad neural systems: the 1) dopamine and related norepinephrine system; 2) serotonin; 3) testosterone; and 4) estrogen and oxytocin system [1]. These proposed temperament dimensions are here named, respectively, 1) Curious/Energetic, 2) Cautious/Social Norm Compliant, 3) Analytical/Tough-minded, and 4) Prosocial/Empathetic. A 56-item questionnaire, the Fisher Temperament Inventory (FTI; Table S1), was developed and then tested using the responses of 34,813 members of a U.S. Internet dating site. Defining personality variables by using broad physiological systems may improve discriminability among individuals and add to the understanding of normal personality structure. In addition, partner compatibility assessment may benefit from a new personality model of four broad dimensions.

Many personality psychologists have proposed models of personality structure [2]–[10]. Many have also theorized about the physiological foundations of their proposed models of temperament [6], [8], [9], [11]–[13]. The Big Five trait constellations are the most widely investigated. Data on the Big Five are now available for countries in Europe, North America and East Asia [14], as well as for several species of birds and other mammals [15], [16], indicating that the Big Five temperament dimensions are widespread in *Homo sapiens* and other species. Moreover, it is now estimated that the Big Five dimensions are largely heritable, with estimates ranging from 40 to 50 percent heritability [17]. Recently, researchers using MRI have begun to correlate the Big Five (NEO-Five Factor Inventory) scale scores [5] with size of brain regions or functional responses [18]–[21]. These studies provide explanatory biological constructs for the Big Five psychological traits, which had previously been determined by behavioral factors. The present studies use regional neural responses to begin to discover biological constructs for the FTI. The brain’s functional response has advantages over measuring peripheral levels of transmitters or hormones, because it shows that an effective influence is present.

To investigate whether the FTI measures brain activity affected by four broad neural systems, the questionnaire was administered as part of two functional magnetic resonance imaging studies (fMRI). During the brain scanning experiments, participants looked at a facial image of their romantic partner and also a familiar, emotionally-neutral individual [22], [23]. Study #1 correlated scores on the FTI and neural activation specific to a partner in a long-term relationship [22]. Study#2 correlated scores on the FTI and neural activation specific to a partner in a pre-marital (engaged) or newlywed relationship [23]. We used this task to test the FTI because it is part of our ongoing research program to determine the neural systems that influence romantic and long-term relationships. It is reasonable to assume that temperament dimensions are revealed under many task conditions, including one that involves thinking about a romantic partner, a near-universal human experience. We were especially interested to determine if there could be a unique “neural signature” for each dimension associated with close, love relationships. The questionnaire was originally tested on a large Internet dating site population of people looking for a romantic partner.

Scores on the Curious/Energetic scale were predicted to correlate with activation in brain regions associated with dopamine systems and dopamine-associated behaviors. Scores on the Cautious/Social Norm Compliant scale were predicted to correlate with activation in regions associated with social norm compliant behaviors. The Analytical/Tough-minded and Prosocial/Empathic scale scores were predicted to correlate with activation in brain regions associated with behaviors linked to sex hormones. The studies provide evidence that the FTI might measure influences of dopamine and sex hormones on local brain responses to romantic partners.

## Methods

### Ethics Statement

Study #1 was approved by the Institutional Review Boards at Stony Brook University and New York University (approval number 6139). Study #2 was approved by the Institutional Review Boards at the University of California, Santa Barbara and Albert Einstein College of Medicine (approval number 2008–418). All participants provided written informed consent and received payment for their participation.

### Participants

#### Study #1

Participants were 17 (10 women) healthy, right-handed individuals ages 39 to 67 (*M* = 52.85, *SD* = 8.91) who self-reported being happily married a mean of 21.4 years (*SD = *5.89) to an opposite-sex partner. Participants were recruited by word-of-mouth, flyers and newspaper ads in the New York Metropolitan area. Individuals were screened by phone for eligibility criteria, including relationship criteria, right-handedness, non-use of antidepressants and fMRI contra-indications. Data on these participants have been published previously [22].

#### Study #2

Participants were 18 (10 women) healthy, right-handed individuals ages 21 to 32 (*M* = 27.50, *SD* = 3.13) in pre-marital (engaged) and newlywed partnerships (mean 4.30 years; *SD* = 3.18). Subjects were recruited by newspaper and Internet ads and flyers as part of a larger study in the Santa Barbara community. Individuals were screened by phone for eligibility criteria, including being married to or about to be married to a first-time spouse, age (22–40), relationship length (<7 years), non-use of anti-depressants and fMRI contra-indications.

### Materials

#### Questionnaire

Participants completed the FTI (Table S1), a 56-item questionnaire that was originally developed and tested using a factor analysis on 39,913 participants on Chemistry.com (a subsidiary of Match.com), an internet dating site [1], [24]. There were 14 statements to measure traits in each of four trait constellations. Each statement had four response options: “strongly disagree,” “disagree,” “agree,” and “strongly agree.” The Curious/Energetic scale included statements such as, “I am always doing new things,” “My friends would say I am very curious,” and “I have more energy than most people.” (scale alpha: Study#1 = 0.84, Study#2 = 0.90). The Cautious/Social Norm Compliant scale included statements such as: “People should behave in ways that are morally correct,” “My friends and family would say I have traditional values,” and “In general, I think it is important to follow rules.” (scale alpha: Study#1 = 0.87, Study#2 = 0.80). The Analytical/Tough-minded scale included statements such as: “I enjoy competitive conversations,” “I am more analytical and logical than most people,” and “I understand complex machines easily” (scale alpha: Study#1 = 0.82, Study#2 = 0.81). The Prosocial/Empathetic scale included statements such as: “I like to get to know my friends deepest needs and feelings,” “I highly value deep emotional intimacy in my relationships,” and “Regardless of what is logical, I generally listen to my heart when making important decisions” (scale alpha: Study#1 = 0.83, Study#2 = 0.88).

### Stimulus Presentation Protocol, Study #1 and Study #2

Procedures are described in detail in Acevedo et al., [22]. The stimuli were presented during a 12-minute session using a block design. Participants viewed two alternating face images interspersed with a count-back task for 20-seconds each, with 6 repetitions. The countback task consisted of counting backwards from a large number like 8011 on the screen, and was used to reduce carry-over effects as well as a control for arousal and attention, replicating procedures in Aron et al. [25].

### Face Stimuli, Instructions, Post-scan Interviews and Facial Attractiveness

Color photographs of facial stimuli, provided by participants prior to scanning, were digitized according to standard procedures and shown using Presentation software (Psychological Software Tools, Inc., Pittsburgh, PA). The Positive Partner (P) stimulus photo was of the long-term spouse or pre-marital/newlywed partner. To control for human face activations and familiarity, we used a photo of a Highly-Familiar Neutral (HFN) acquaintance matched for gender, age and length of time known to the participant. Participants were instructed to think about romantic experiences with the partner that were not sexual in nature to control for event memory, neutral experiences with the HFN acquaintance. After each set of images, while still in the scanner, participants rated the emotional intensity elicited by each stimulus. These data are presented in Acevedo et al. [22] Post scan interviews were conducted to assess whether instructions were followed. Participants were asked to describe their thoughts and feelings during the experiment, and whether they were able to do the count-back task. Also, all photos were rated for attractiveness and image quality by six independent raters and there were no differences between P and HFN faces. For details see Acevedo et al. [22]. These procedures have been used in four separate fMRI studies carried out by our group [22], [25]–[27], and by others [28]–[31]. They result in replicable activations associated with romantic love and attachment.

### Data Acquisition

#### Study #1

The scanning procedures have been published previously [22]. Briefly, data were acquired with a 3T Siemens magnetic resonance imaging system located in the Center for Brain Imaging at New York University. A repetition time (TR) of 2,000-ms was used, with a TE of 30-ms, a 90° flip angle, and a voxel size for functional images of 3×3×3 mm.

#### Study #2

MRI scanning was performed using a 3T Siemens magnetic resonance imaging system with a NOVA head coil at the Brain Imaging Center of the University of California, Santa Barbara. Anatomical scans were obtained first. Next, functional images were obtained. The first four volumes were discarded to allow for proper calibration, resulting in 360 functional images, in volumes of 30 slices consisting of 3-mm thick axial slices (0 mm gap) covering the whole brain. A TR of 2,000-ms was used with a TE of 30-ms, a 90° flip angle, and a voxel size for functional images of 3×3×3 mm.

### Data Analysis

For Study #1 [22], data were analyzed using SPM2; for Study #2, data were analyzed using SPM5 (http://www.fil.ion.ucl.ac.uk/spm). For preprocessing, functional EPI volumes were realigned to the first volume, smoothed with a Gaussian kernel of 6 mm, and then normalized to the MNI T1 image template. No participant showed movement greater than 3 mm (whole voxel) motion. After preprocessing, analyses were carried out using a mixed effects general linear model, with participants as the random-effects factor and conditions as the fixed effect. The P-versus-HFN contrast was created.

#### Correlations: positive vs. highly familiar neutral contrast

Simple regression analyses of the four scale scores (i.e., Curious/Energetic, Cautious/Social Norm Compliant, Analytical/Tough-minded, and Prosocial/Empathetic scores) with brain activations were applied to the P-versus-HFN contrast. Exploratory whole-brain analyses were conducted, applying a threshold of *p*≤.001 (uncorrected for multiple comparisons) with a spatial extent of ≥15 contiguous voxels. Results of the whole-brain exploratory analysis are indicated in the Tables with superscripts. Region of interest (ROI) analyses, i.e. planned comparisons, were carried out to examine activations in dopamine-related areas for the Curious/Energetic scale; in the ventrolateral prefrontal cortex for the Cautious/Social Norm Compliant scale [32]; and in the inferior frontal gyrus, fusiform and insular cortex for the Prosocial/Empathic scale [33], [34]. For ROIs, a false discovery rate (FDR) was used for multiple comparisons correction [35] with a threshold of *p*≤.05. Results of this analysis are indicated by superscripts in the Tables. Results from Study #1 were used for ROIs in Study #2. The ROIs occupied a 3–5 mm radius. Anatomic regions were confirmed with an atlas of the human brain [36].

## Results

### Replicated Results

**Figure 1:**
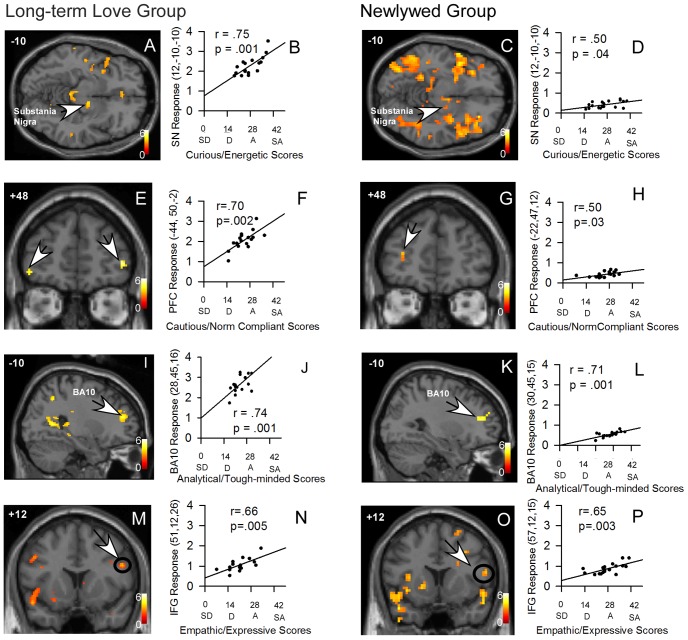
Localization of correlations with the Fisher Temperament Inventory scores. Brain images show regional activation correlated with the questionnaire scores for the four temperaments. Graphs show the correlation associated with the region indicated by an arrow in the image on its left. Peak locations were the same or within 10**A.–D.** The dopamine-rich substantia nigra region activation (arrows), shown in an axial image, was correlated with Curious/Energetic scores. **E.–H.** The ventrolateral prefrontal cortex activation (arrows), shown in a coronal image, was correlated with Cautious/Social Norm Compliant scores. The two groups showed slightly different activation areas, but both were associated with social norm compliance in another study (ref). **I.–L.** The dorsolateral prefrontal cortex activation (arrows, BA10), shown in a sagittal image, was correlated with Analytical/Tough-minded scores. **M.–P.** The inferior frontal gyrus activation (arrows), shown in a coronal image, was correlated with Empathic/Expressive scores. The Long-Term Love experiment was done first, and regions of interest for the Newlywed experiment analysis were based on it. BA10, Brodmann’s Area 10, dorsolateral prefrontal cortex. IFG, inferior frontal gyrus. PFC, prefrontal cortex, ventrolateral. SN, substantia nigra. The color scale shows t-scores. MNI template: right in the image is right side of brain. Graphs x axes: SD, strongly disagree; D, disagree; A, agree; SA, strongly agree.

For the Long-Term Love group, Study #1, scores on the Curious/Energetic scale of the FTI co-varied with activations in the region of the right substantia nigra (SN: r = .75, p = .001) and right dorsolateral prefrontal cortex (DLPFC, BA10: r = .74, p = .001) (Figure 1A–B, Table 1). These results were replicated in Study #2, the Newlywed group (SN: r = .50, p = .04; DLPFC: r = .71, p = .001; Figure 1C–D; Table 1). Also, a small area of the auditory cortex (BA22) and the SI gustatory area (BA43) were correlated with Curious/Energetic scores in both Study #1 (BA22: r = .79, p = .001; BA43: r = .82, p = .001) and Study#2 (BA22: r = .79, p = .001; BA43: r = .80, p = .001), but in different regions (Table 2).

**Table 1:** Brain regions show positive correlations between the Fisher Temperament Inventory Scores and neural activation in two independent studies within the Positive>Neutral contrast.

Brain region	Long-term Love Group				Newlywed Love Group			
	x	y	z	P	x	y	z	P
**Curious/Energetic**								
Substantia Nigra^1^	+12	−10	−10	.002	+15	−9	−9	.046
Dorsolateral prefrontal cortex^5^	+22	+56	+18	.001	+36	+57	+21	.006
**Cautious/Social Norm Compliant**								
Ventrolateral prefrontal cortex^2^	−44	+50	+2	.001	−22	+47	+12	.015
**Analytical/Tough-Minded**								
Occipital cortex^4^	−14	−82	18	<.001	−15	−75	15	.008
Occipital cortex (BA18) 4	−4	−78	28	.001	−3	−78	28	.028
Parietal cortex (BA7) 4	+2	−58	+48	.001	+9	−58	+51	.005
Dorsolateral prefrontal cortex (BA 10) 4	+28	+45	+16	.001	+30	+45	+15	.003
Dorsomedial prefrontal cortex	−16	+40	+28	.001	−18	+39	+36	.029
	+25	+46	+18	.001	+25	+45	+18	.007
Orbitofrontal cortex^4^	+42	+48	−2	.001	+42	+48	−3	.012
**Prosocial/Empathic**								
Inferior frontal gyrus^3^	+52	+14	+26	.025	+57	+12	+14	.005
Anterior insula^3^	−26	+26	−10	.003	−27	+27	−12	.012
Fusiform gyrus	−32	−37	−18	.007	−33	−48	−15	.002

Regions of interest analysis noted by superscripts. MNI coordinates (x,y,z) are at the maximum value for the cluster, which may be elongated in any direction.

1 We searched dopamine and norepinephrine-rich regions.

2 From Spitzer et al., [32]: both regions in the two groups were correlated with social norm compliance.

3 From refs [33].

4 Exploratory analysis.

**Table 2:** Brain regional correlations between scores for personality dimensions and neural activation that were unique to the two groups tested (within Positive>Neutral contrast).

Brain region	Long-Term Love				Newlywed Love			
	x	y	z	P	x	y	z	P
**Curious/Energetic**								
Locus Coeruleus region^1^					−6	−30	−27	.046
BA22 (auditory)^2^	−46	−10	−7	.001	+57	−6	−3	<.001
BA43 (somatosensory/gustatory)^2^	−60	−10	20	.001	−48	+3	+15	<.001
**Cautious/Social Norm Compliant**								
Posterior hypothalamus^2^	+4	−4	−8	.001				
Angular gyrus^2^	−50	−56	+52	.001				
	−39	−74	+39	.001				
Prefrontal cortex, ventrolateral^2^	+45	+50	+2	.001				
Parietal cortex, lateral^2^	−38	−74	+44	.001				
	+36	−76	+44	.001				
**Analytical/Tough-Minded**								
Hippocampus^2^	+36	−30	−6	.001				
**Prosocial/Empathetic**								
Retrorubral field, midbrain^1^					+6	−18	−15	.003
Orbitofrontal cortex^1^	−35	+29	−16	.008				

MNI coordinates (x,y,z) are at the maximum value for the cluster, which may be elongated in any direction.

1 We searched dopamine and norepinephrine-rich regions.

2 Exploratory whole brain analysis.

Scores on the Cautious/Social Norm Compliant scale correlated with activation in the left ventrolateral prefrontal cortex (vlPFC) in Study #1 (r = .70; p = .002; Figure 1E–F) and in Study #2 (r = .50; p = .03; Figure 1G–H; Table 1).

Scores on the Analytical/Tough-minded scale co-varied with activation in the occipital (BA18) and parietal cortex (BA7) in Study #1 (BA18: r = .71, p = .001; BA7: r = .79, p = .001) and Study#2 (BA18: r = .59, p = .01; BA7: r = .63, p = .01). Scores also co-varied in both studies with activation in an area of the right DLPFC (BA10) in Study# 1(r = .74, p = .001) and Study# 2 (r = .71, p = .001), although regionally different from that associated with the Curious/Energetic scale; in a region of the right and left dorsomedial prefrontal cortex (DMPFC) in Study# 1(right: r = .70, p = .002; left: r = .78, p = .001) and Study#2 (right: r = .55, p = .02; left: r = .56, p = .02); and a region of the right orbitofrontal cortex (rOFC) in Study #1 (r = .78, p = .001) and Study#2 (r = .60, p = .01). See Table 1.

Scores on the Prosocial/Empathetic scale correlated with activation in the right inferior frontal gyrus (IFG) in Study#1 (r = .66, p = .005) and Study# 2 (r = 65; p = .003); the left anterior insula (AI) in Study#1 (r = .57, p = .02) and Study# 2 (r = .54, p = .02); and the left fusiform gyrus in Study# 1 (r = .62, p = .007) and Study#2 (r = .64, p = .004). See Table 1.

### Results Unique to Each Study Group

#### Study #1, the long-term love sample

The whole brain, exploratory analysis showed several unique regions of correlated activation (Table 2). The Cautious/Social Norm Compliant scale co-varied with activation of the posterior hypothalamus (r = .68, p = .001); the left angular gyrus (r = .76, p = .001); the right vlPFC (r = .71, p = .001); and left and right lateral parietal cortex(r = .71, p = .001; r = .69, p = .001, respectively). Scores on the Analytical/Tough-minded scale showed a correlation with activation in the right hippocampus (r = .78, p = .001). For regions of interest, scores on the Prosocial/Empathic scale co-varied with activation of the left OFC (r = .57, p = .02). For localization results see Table 2.

#### Study #2, the pre-marital and newlyweds sample

For regions of interest, the norepinephrine-rich locus coeruleus was correlated with scores on the Curious/Energetic scale (r = .57, p = .01). A left vlPFC area showed activation in association with scores on the Cautious/Social Norm Compliant scale (r = .51, p = .03). Activation in a midbrain area of the retrorubral field was correlated with scores on the Prosocial/Empathic scale (r = .63, p = .003). For localization results see Table 2.

### Scale Scores for the Four Dimensions

Both groups showed an adequate range of scores for each dimension, from 15 to 35 out of a possible range from 0–42 (Figure 1). Thus, the scores included answers from Disagree through Agree, to nearly Strongly Agree.

Overall, in these two groups of volunteer participants, 8 showed highest scores on the Curious/Energetic dimension, 11 showed highest scores on the Cautious/Social Norm Compliant dimension, 4 showed highest scores on the Analytical/Tough-Minded dimension and 12 showed highest scores on the Prosocial/Empathic dimension.

### Blood-Oxygen-Level Dependent (BOLD) Response

The brain’s physiological BOLD response (parameter estimates calculated by SPM) was not 0 or negative at the lowest scores for any of the temperament dimension correlations (Figure 1). All responses were positive, although some more than others, producing the positive correlation.

## Discussion

The objective of the analysis of two fMRI studies was to identify any neural regions and systems associated with four broad temperament dimensions measured by the Fisher Temperament Inventory [1] in people in love. Also, an objective was to test the hypothesis that these four temperament dimensions are associated with influences of dopamine/norepinephrine, serotonin, testosterone and estrogen/oxytocin in the brain. The results showed that scores on each of the four FTI scales did correlate with activations in some predicted brain regions. The case of the Curious/Energetic scores correlation with the region of the substantia nigra (SN) is the strongest evidence for involvement of the predicted transmitter system. The case of the Cautious/Social Norm Compliant dimension’s association with the serotonin system is the weakest. No brainstem regions rich in serotonin cells, nor forebrain regions especially rich in serotonin receptors were associated with the Cautious/Social Norm Compliant dimension. But a brain region containing serotonin receptors and associated with “social norm compliance” in other fMRI studies was correlated with that dimension. The correlations between Analytical/Tough-minded and Prosocial/Empathic with brain regions influenced by sex hormones is indirect (see below), but functional, evidence in support of the hypothesis. The findings were replicated in two separate studies, making them highly significant. The data support the hypothesis that the FTI measures specific transmitter and hormonal influences in the brain.

### Curious/Energetic Scale

The SN is a major group of cells in the dopamine system [37]–[41] where dopaminergic influences can be expected. Scores on the Curious/Energetic scale of the FTI co-varied with activation in the exact same region of the SN in both Study #1 and Study #2. Although dopamine activity was not directly monitored in this study, the SN is rich with dopamine cells and receptors, and its activation can have widespread effects on behavior through dopamine’s actions [37]–[41]. The replication of activation in this region is strong evidence that the Curious/Energetic scale of the FTI measures activity involving the dopamine system more than the other scales. Although this temperament dimension certainly uses other transmitter systems for this task, the dopamine system may be a primary influence. It is important to note that this is a region different from the dopamine-rich ventral tegmental area that is activated in most participants in love regardless of personality [22], [25]–[27].

For the younger pre-marital and newlywed group, the norepinephrine-rich region of the locus coeruleus was also correlated with scores on the Curious/Energetic scale. This is additional support for the idea that the dopamine and norepinephrine systems are important physiological correlates of the Curious/Energetic temperament dimension. Extraversion, one of the Big Five traits that has been associated with high energy has also been associated with the dopamine system [12].

### Cautious/Social Norm Compliant Scale

Scores on the Cautious/Social Norm Compliant scale co-varied with activation in regions of the left vlPFC in both Study#1 and Study #2. The two vlPFC regions in both studies were associated with “social norm compliance” behavior in a previous study [32], and social norm compliance is linked with activity in the serotonin system [20], [42]. Although there are many neurotransmitter and receptor types in the vlPFC, serotonin is one of them [43], [44]. These results are indirect evidence that the Cautious/Social Norm Complaint scale of the FTI could measure activity associated with the brain’s serotonin system.

### Analytical/Tough-minded Scale

In both studies, scores on the Analytical/Tough-minded scale correlated with activation in primary areas of the occipital cortex, which mediates basic visual functions. These results are consistent with the prediction that this temperament dimension is influenced by testosterone, as suggested by several studies. In one study, men were more sensitive than women in a visual contrast sensitivity task; men showed greater visual acuity for detail and rapidly moving stimuli [45]. Endogenous testosterone is associated with enhanced attention to visual details [46]. Also, in an fMRI study, light stimulation had a greater effect on the occipital cortex in men than in women [47], and men excel at seeing in the light [48]. Animal studies have shown that, during development, males have a larger number of androgen receptors in the occipital cortex than do females [49], and androgen receptors persist in these cortical regions in adult primates, including the visual cortex [50]. Equally relevant, using magnetic resonance imaging to measure tissue density, anatomical studies have found sex differences in the occipital cortex [51], [52]. Thus, the occipital cortex is an area where sex differences have been documented functionally and anatomically, and the positive correlation between the Analytic/Tough-minded temperament dimension and occipital cortex activation is evidence that the Analytical/Tough-minded scale may measure some aspect of testosterone system activity.

In addition, areas of the parietal cortex correlated with scores on the Analytical/Tough-minded scale in both studies. The parietal cortex is involved in spatial/mathematical thinking, and anatomical studies show sexual dimorphism in this region associated with male/female differences in spatial/mathematical tasks [51], [53]–[55]. The parietal regions identified in these other studies were within 10 mm of the regions where a correlation was found with the Analytical/Tough-minded dimension. General measurements of parietal lobe structure and function also show male/female differences [56]–[58]. Further, prenatal endogenous testosterone priming has been linked with enhanced visual-spatial perception and mathematical skills [59]–[61]. Activation of these parietal regions, as well as activation in the DLPFC, may reflect the “analytic” aspect of the proposed Analytical/Tough-minded temperament dimension [47], [49]–[58], [62], [63].

Regions of the DLPFC, DMPFC and OFC also correlated with the Analytical/Tough-minded scale. These are areas involved in cognition and reward assessment e.g. [64]–[73].

The DLPFC, DMPFC, OFC and each of the specific regions correlated with the Analytical/Tough-minded dimension are within 10 mm of an area that discriminated between men and women in anatomical measurement studies of regional gray matter size and density [51], [52], [62]. Thus, all regions correlated with the Analytical/Tough-minded temperament dimension have shown anatomical differences between men and women, strongly suggesting a hormonal influence [47], [49]–[58], [62].

### Prosocial/Empathetic Scale

Scores on the Prosocial/Empathetic scale correlated in both studies with activity in the IFG, AI and fusiform gyrus. These areas are associated with mirror neurons or empathy [33], [34]. Empathy is regularly associated with estrogen activity. Moreover, each of these regions is associated with sex differences, sometimes directly attributed to estrogen activity. Witte et al., 2010 [74] found salivary 17beta-estradiol to be associated with gray matter volume in the fusiform gyrus in a region close to the region correlated with scores on the Prosocial/Empathetic scale of the FTI (within 10 mm). They also found a correlation for 17beta-estradiol in the IFG on the other side of the brain, which correlated with the Prosocial/Empathetic scale of the FTI [74]. Cheng et al., 2009 [75] found greater gray matter volume in women compared to men in the pars opercularis, near the IFG region that correlated with Prosocial/Empathetic scores in the present study. Cheng et al, 2009 [76] also found a correlation between gray matter volume and empathy measures in men and women in the pars opercularis. Using a multimodal approach, Feis et al. [51] report differences between men and women in brain tissue (volume, density) in the AI and IFG cortex. Last, Yamasue et al., 2008 [76] found greater gray matter volume in the IFG in women; these women also showed greater cooperativeness than the men in the study. These data from several sources support the prediction that the Prosocial/Empathetic scale of the FTI is measuring influences of the estrogen system.

### Study Replication

The replication of the results in two separate studies indicates that the effects are reliable. The results show an association with a dopamine-rich brain region, as well as testosterone and estrogen influences documented in many other studies. Thus, one out of the four dimensions was directly associated with the predicted biological system, and the two sex hormone-based dimensions were associated with sex hormone structural and functional effects in the brain. Moreover, the Cautious/Social Norm Compliant dimension was associated with predicted regions based on behavior, and serotonin could certainly be involved.

The replication of these results in different age groups also shows the robustness of the dimensions over the life course. The subjects in Study #1 ranged in age from 39 to 67 (*M* = 52.85) and were in marriages of considerable duration (M = 21.4 years). Subjects in Study #2 ranged in age from 21 to 32 (*M* = 27.5) and were in pre-marital or newlywed relationships of far shorter duration (M = 4.3 years). Other research shows that a range of personality variables are relatively stable over the life course [77]–[80], and we predict that the four dimensions reported here will be consistent within individuals, also.

### Results Unique to Each Group

A few correlations were found in only one of the two groups studied. Of special note, a correlation with activation in the locus coeruleus region, rich in norepinephrine-producing cells, was found for the Curious/Energetic scale of the Pre-marital/Newlywed group. This association is predicted by the FTI model. But it is not known why this correlation appeared in one group and not the other. A range of factors may be involved, including variations in mean age and composition of each group, variations in the degree of feelings of romance and attachment expressed by participants in each group, and/or a difference in scanner sensitivity.

Also regarding the Curious/Energetic scale, there was a correlation with the auditory cortex; but the two groups show this activity on different sides of the brain. The two groups also showed a correlation between the somatosensory/gustatory area and scores on the Curious/Energetic scale of the FTI, but these activations were farther away from each other than our criterion for replication would allow. Nevertheless, these correlations suggest that auditory and taste sensations may be especially important to those individuals expressive of traits associated with the Curious/Energetic dimension. Indeed, sensation-seeking individuals score high on scales that measure sensation-seeking through the mind and senses [81]; these individuals prefer arousing sensory stimuli in the arts [82], music [83], live entertainment [84] and food preferences [85]. Perhaps the subjects who were primarily expressive of the Curious/Energetic scale of the FTI had more arousing auditory and sensory/gustatory-related thoughts and memories about their partner while doing the task.

### Limitations and Future Directions

To explore the full complement of biological processes associated with the trait constellations measured by the FTI, several types of studies need to be designed and implemented. Although investigations of genetic markers related to the dopamine and serotonin systems are in progress, it may take decades before genome-wide association studies will be able to measure the full array of genes that contribute to any of these proposed trait constellations. Besides genetic studies, direct measurement of hormones in saliva is possible, and positron emission tomography could be used to confirm specific transmitter involvement in the four dimensions proposed here. However, there is an advantage to seeing the functional associations of fMRI before embarking on direct and sometimes invasive measurements of the transmitters and hormones directly. For hormone levels, especially, which vary widely throughout the day, it is better to have a functional measure of the effect the hormone has had, such as regional brain size and density variations and regional activation correlations, before undertaking direct level measurements.

Further, the current FTI and its scoring describes only four major trait constellations. Future investigations may uncover lower level trait constellations, as well as expose the highly complex relationships among these temperament dimensions and with other dimensions of temperament.

In addition, Study#1 and Study#2 investigated brain activity while participants did only one task. Additional brain scanning studies (fMRI) of different groups and different tasks must be conducted. Interestingly, the local brain response was never zero or negative for a region correlated with one of the dimensions (Figure 1). All participants showed some response in the region where a significant correlation was found, suggesting a general “social response” to a romantic partner in each region, but greater for some temperament dimensions than for others. This suggests that the findings are a “social response signature” for each dimension, as we sought to determine. There might be a different set of regions involved for each dimension if the task had been a mathematical one, for example. Importantly, the four FTI subscales produced unique high activations in separable brain regions; we predict that these temperament dimensions will be discriminable in different kinds of tasks.

### Conclusion

Scores on the Curious/Energetic scale of the FTI co-varied with activation in a region of the substantia nigra in two independent studies, providing strong evidence that the Curious/Energetic scale could measure some aspect of the dopamine system activity in people thinking about their romantic partner. Scores on the Cautious/Social Norm Compliant scale co-varied in both studies with activation in the ventrolateral prefrontal cortex, in a region associated with “social norm compliance,” a trait linked in the biological literature with the serotonin system, indirect evidence that the Cautious/Social Norm Compliant scale measures some aspect of the serotonin system. Scores on the Analytical/Tough-minded scale co-varied in these two studies with activity in regions of the occipital, parietal, orbitofrontal and prefrontal cortex, regions affected by sex hormones and associated with sex differences in behavior. Last, scores on the Prosocial/Empathetic scale correlated in both studies with activity in regions associated with mirror neurons and concomitant empathy, a trait linked with the estrogen system, and brain regions structurally affected by gender. Although each of the temperament dimensions use many other neurochemical systems, one or two appear to predominate in each dimension under these specific task circumstances.

These findings support the hypothesis that the four broad temperament dimensions measured by the FTI are associated with separable brain systems. Because the results were replicated in two independent studies using participants of significantly different ages, these data also suggest that traits associated with these four temperament dimensions may be relatively stable across the life course. Finally, the results suggest that there could be a unique “neural signature” for each temperament dimension associated with close, love relationships.

## Supporting Information

Table S1 **Fisher Temperament Inventory and Tie Breakers.** Each question received a score of 0–3 for Strongly Disagree, Disagree, Agree and Strongly Agree.
(DOCX)
