# Supplementary material for: Neural Correlates of Four Broad Temperament Dimensions: Testing Predictions for a Novel Construct of Personality
Source: PLoS One. 2013 Nov 13;8(11):e78734. doi: 10.1371/journal.pone.0078734 (PMC3827248; doi:10.1371/journal.pone.0078734)
Supplement: Table S1 — Fisher Temperament Inventory and Tie Breakers. Each question received a score of 0–3 for Strongly Disagree, Disagree, Agree and Strongly Agree. (DOCX) [file pone.0078734.s001.docx]

| **Curious/Energetic** | |
| --- | --- |
| 1 | I find unpredictable situations exhilarating. |
| 2 | I do things on the spur of the moment. |
| 3 | I get bored when I have to do the same familiar things. |
| 4 | I have a very wide range of interests. |
| 5 | I am more optimistic than most people. |
| 6 | I am more creative than most people. |
| 7 | I am always looking for new experiences. |
| 8 | I am always doing new things. |
| 9 | I am more enthusiastic than most people. |
| 10 | I am willing to take risks to do what I want to do. |
| 11 | I get restless if I have to stay home for any length of time. |
| 12 | My friends would say I am very curious. |
| 13 | I have more energy than most people. |
| 14 | On my time off, I like to be free to do whatever looks fun. |
| **Cautious/Social Norm Compliant** | |
| 15 | I think consistent routines keep life orderly and relaxing. |
| 16 | I consider (and reconsider) every option thoroughly before making a plan. |
| 17 | People should behave according to established standards of proper conduct. |
| 18 | I enjoy planning way ahead. |
| 19 | In general, I think it is important to follow rules. |
| 20 | Taking care of my possessions is a high priority for me. |
| 21 | My friends and family would say I have traditional values. |
| 22 | I tend to be meticulous in my duties. |
| 23 | I tend to be cautious, but not fearful. |
| 24 | People should behave in ways that are morally correct. |
| 25 | It is important to respect authority. |
| 26 | I would rather have loyal friends than interesting friends |
| 27 | Long established customs need to be respected and preserved. |
| 28 | I like to work in a straightforward path toward completing the task. |
| **Analytical/Tough-minded** | |
| 29 | I understand complex machines easily. |
| 30 | I enjoy competitive conversations. |
| 31 | I am intrigued by rules and patterns that govern systems*.* |
| 32 | I am more analytical and logical than most people. |
| 33 | I pursue intellectual topics thoroughly and regularly. |
| 34 | I am able to solve problems without letting emotion get in the way |
| 35 | I like to figure out how things work. |
| 36 | I am tough-minded. |
| 37 | Debating is a good way to match my wits with others. |
| 38 | I have no trouble making a choice, even when several alternatives seem equally good at first. |
| 39 | When I buy a new machine (e.g., camera, computer or car), I want to know all of its technical features. |
| 40 | I like to avoid the nuances and say exactly what I mean. |
| 41 | I think it is important to be direct. |
| 42 | When making a decision, I like to stick to the facts rather than be swayed by people’s feelings. |
| **Prosocial/Empathetic** | |
| 43 | I like to get to know my friends’ deepest needs and feelings. |
| 44 | I highly value deep emotional intimacy in my relationships. |
| 45 | Regardless of what is logical, I generally listen to my heart when making important decisions. |
| 46 | I frequently catch myself daydreaming. |
| 47 | I can change my mind easily. |
| 48 | After watching an emotional film I often still feel moved by it several hours later. |
| 49 | I vividly imagine both wonderful and horrible things happening to me. |
| 50 | I am very sensitive to people’s feelings and needs. |
| 51 | I often find myself getting lost in my thoughts during the day. |
| 52 | I feel emotions more deeply than most people. |
| 53 | I have a vivid imagination. |
| 54 | When I wake up from a vivid dream, it takes me a few seconds to return to reality. |
| 55 | When reading, I enjoy when the writer takes a sidetrack to say something beautiful or meaningful. |
| 56 | I am very empathetic. |

| **Tied Types** | | **Tiebreaker Questions** |
| --- | --- | --- |
| 1. | Curious/Energetic | I constantly seek new adventures. |
|  | Cautious/Norm Compliant | I generally prefer to do familiar things. |
| 2. | Curious/Energetic | I’m interested in all kinds of different people. |
|  | Analytic and Tough-Minded | I am interested in people who share my deepest interests. |
| 3. | Curious/Energetic | I’m not very introspective; I like to look out not in |
|  | Prosocial/Empathetic | I’m very introspective; I’m interested in deeply understanding others |
| 4. | Cautious/Norm Compliant | I tend to be cautious in my work and thinking. |
|  | Analytic and Tough-Minded | I tend to be daring in my work and thinking. |
| 5. | Cautious/Norm Compliant | I tend to think concretely; I only trust the facts. |
|  | Prosocial/Empathetic | I tend to be imaginative and listen to my intuition. |
| 6. | Analytic and Tough-Minded | I tend to be tough minded. |
|  | Prosocial/Empathetic | I tend to be tender hearted. |
